# Recent advances and perspectives in ruthenium-catalyzed cyanation reactions

**DOI:** 10.3762/bjoc.18.4

**Published:** 2022-01-04

**Authors:** Thaipparambil Aneeja, Cheriya Mukkolakkal Abdulla Afsina, Padinjare Veetil Saranya, Gopinathan Anilkumar

**Affiliations:** 1 School of Chemical Sciences, Mahatma Gandhi University, PD Hills, Kottayam, Kerala, 686560, India

**Keywords:** cyanation, nitriles, photocatalyst, ruthenium, tertiary amines

## Abstract

The cyanation reaction has achieved rapid progress in recent times. The ability to exhibit multiple oxidation states increased the demand of ruthenium in the field of catalysis. These cyanation reactions have wide application in pharmacological and biological fields. This review gives an overview of the ruthenium-catalyzed cyanation reactions covering literature up to 2021.

## Introduction

Nitriles are a major class of organic compounds having wide significance in materials science, agrochemical and pharmaceutical industry [1]. They are the privileged compounds finding broad applications in natural product synthesis, pigments and dyes.

Different variety of α-amino carbonyls [2], α-amino acids [3] and 1,2-diamines were prepared from the nitriles using homogeneous and heterogeneous catalysis [4–6]. One of the astonishing aspects of nitriles is that it can be easily converted to amines, carboxylic acids, tetrazoles, aldehydes, amidines, and amides [7–11]. This has been suitably transformed into structurally diverse and complex molecules.

In 1927, Pongratz reported a method towards cyanation reactions [12]. From then, onwards, cyanation gained prime focus and achieved much acceptance. The conventional approaches towards the synthesis of nitriles include Rosenmund–von Braun reactions, Sandmeyer reactions and industrial ammoxidation reactions [13–14]. But these strategies involve harsh reaction conditions and generation of large amount of heavy metal waste. These drawbacks demanded the need of an alternative method for the synthesis of nitriles.

Nowadays transition-metal-catalyzed reactions have received tremendous interest. Various transition metals such as Fe [15], Co [16], Ni [17], Pd [18], Cu [19], Rh [20] etc. were well explored in cyanation owing to its cost-effective and earth abundant characteristics. Moreover, much greener methodologies like microwave-assisted cyanation reactions also received much attention in recent times [21].

The cyanation can be carried out using electrophilic and nucleophilic cyanating agents [22]. Usually a cyanation is accomplished via the nucleophilic attack of a CN^−^ at an electrophilic carbon center. But there are some reagents that react as CN^+^ and thus attack the nucleophilic carbon center. Tosyl cyanide [23], 2-chlorobenzylthiocyanate [24], and cyanogen chloride [25] are some of the examples for electrophilic cyanating agents. Commonly used metallic cyanating agents include K_4_Fe(CN)_6_, CuCN, KCN, NaCN, TMSCN etc.

Ruthenium-catalyzed reactions have gained significant attention in recent times [26]. Ruthenium has the ability to show a large number of oxidation states, and thus a large number of complexes can be prepared using this metal. Ruthenium complexes have astonishing characteristics such as high electron transfer ability, low redox potentials, high Lewis acidity, and greater stabilities of the reactive metallic species like oxometals, metallacycles, and metal carbene complexes [27]. The wide availability of highly reactive ruthenium complexes which are efficient as catalysts elevated the scope of this metal in synthetic organic chemistry.

For clarity and ease of understanding of the topic, this review is categorized into four sections: cyanation of amines, cyanation of arenes and heteroarenes, photocatalyzed cyanation, and miscellaneous. To the best of our knowledge, this is the first review summarizing an overview of ruthenium-catalyzed cyanation reactions covering literature up to 2021.

## Review

### 1 Cyanation of amines

#### 1.1 Cyanation of amines using heterogeneous catalysts

**Scheme 1:**
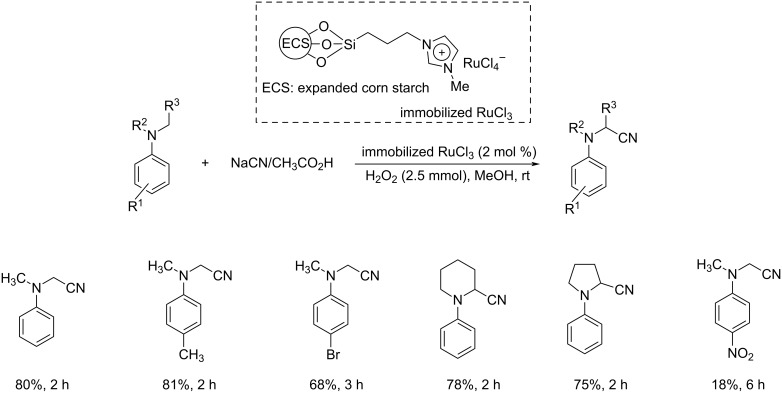
Starch-immobilized ruthenium trichloride-catalyzed cyanation of tertiary amines.

**Scheme 2:**
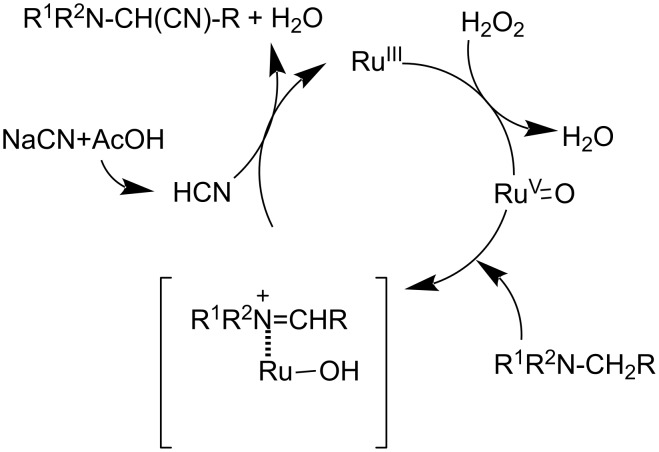
Proposed mechanism for the cyanation of tertiary amines using starch-immobilized ruthenium trichloride as the catalyst.

Sain and co-workers investigated the ability of starch-immobilized ruthenium trichloride to catalyze the oxidative cyanation of tertiary amines [28]. The reaction was performed using 2 mol % of catalyst, 2.5 mmol H_2_O_2_, 1.2 mmol NaCN, and 1 mL CH_3_COOH in methanol at room temperature (Scheme 1). Better yields of the α-aminonitrile products were obtained for aromatic amines with electron-donating substituents than electron-withdrawing ones. Under the optimized conditions, tributylamine was found inactive to afford the desired product. Inductively coupled plasma atomic emission spectroscopy (ICP-AES) analysis confirmed the purity of the prepared compounds without any ruthenium contamination. The attractive features of this reaction include environment-friendly conditions, effective recycling and activity of the catalyst, and high product selectivity. A plausible mechanism of this transformation is depicted in Scheme 2.

**Scheme 3:**
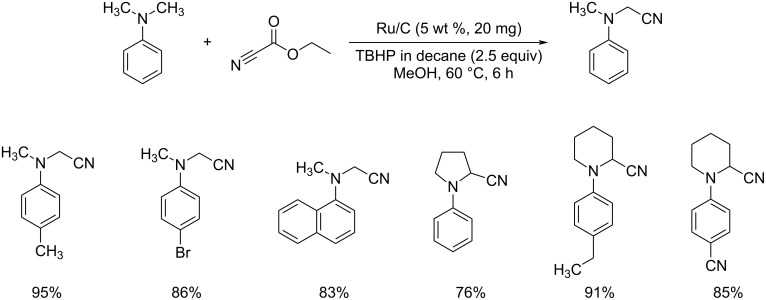
Cyanation of tertiary amines using heterogeneous Ru/C catalyst.

**Scheme 4:**
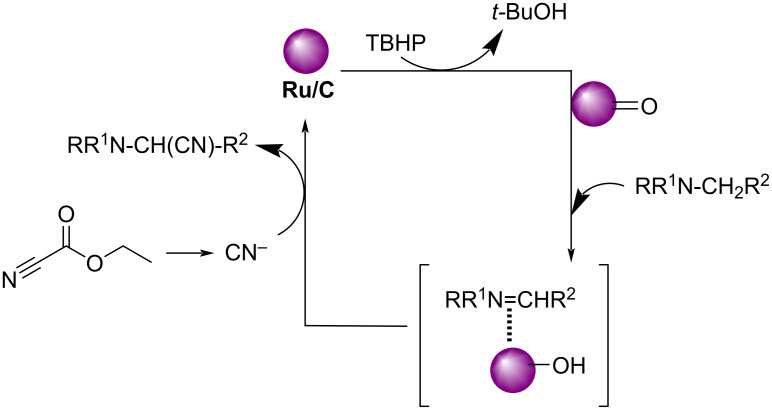
Proposed mechanism for cyanation of tertiary amines using a heterogeneous Ru/C catalyst.

Later in 2012, Nageswar et al. have established a methodology for the oxidative cyanation of tertiary amines using a heterogeneous Ru/C catalyst [29]. Most of the reported works involve the use of toxic cyanating agents like NaCN or TMSCN. In this protocol, the authors have exploited the cost-effective, less toxic, and easily available ethyl cyanoformate as the cyanide source. They carried out the optimization studies using *N,N*-dimethylaniline (1.0 mmol) and ethyl cyanoformate (2.0 mmol) as the model substrates and the optimized conditions include 5 wt % Ru/C (20 mg) as catalyst and 2.5 equiv of TBHP (in decane) as oxidant in methanol at 60 °C for 6 h (Scheme 3). Differently substituted tertiary amines with electron-rich and electron-deficient substituents afforded the required products in good yields. This reaction was found to be suitable for *N*-aryl-substituted cyclic amines such as *N*-arylpiperidine, *N*-arylpyrrolidine, and *N*-aryltetrahydroisoquinoline. However, aliphatic tertiary amines failed to achieve the desired product by this method. The proposed mechanism is initiated with the formation of a Ru–oxo species by the reaction between Ru/C and TBHP. The next step involves the formation of an iminium ion intermediate through reaction of the Ru–oxo species with the tertiary amine. The subsequent reaction between this iminium ion intermediate and CN^−^ furnishes the required product (Scheme 4).

**Scheme 5:**
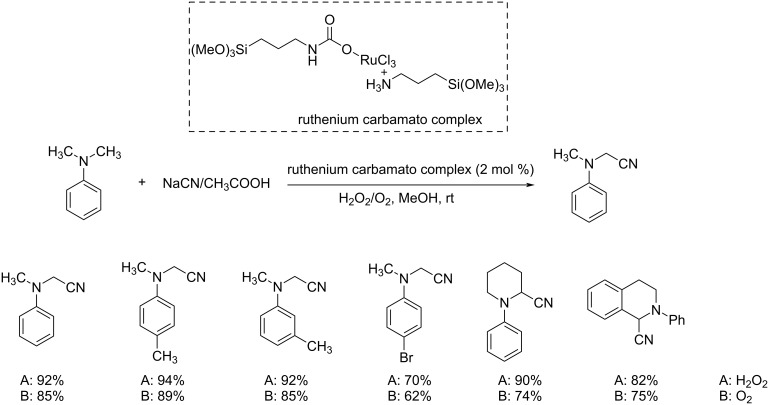
Ruthenium-carbamato complex-catalyzed oxidative cyanation of tertiary amines.

In 2013, Jain and co-workers described a novel strategy for the synthesis of a ruthenium-carbamato complex and its promising catalytic application in the oxidative cyanation reaction [30]. The prepared catalyst was found highly active in the oxidative cyanation of tertiary amines to the corresponding α-aminonitriles in excellent yields. The cyanation reaction of *N,N*-dimethylaniline using NaCN catalyzed by ruthenium-carbamato complex in AcOH/MeOH furnished the desired product in 92% yield within 2.5 h (Scheme 5). Meanwhile, the ruthenium chloride-catalyzed cyanation reaction required 3.5 h to complete the reaction and achieved the product in 90% yield. Thus, the studies revealed the higher efficiency of the ruthenium-carbamato complex in catalyzing the cyanation reaction. This strategy utilized eco-friendly hydrogen peroxide and molecular oxygen as the oxidant system. This method was found highly favorable to tertiary amines with electron-donating substituents.

**Scheme 6:**
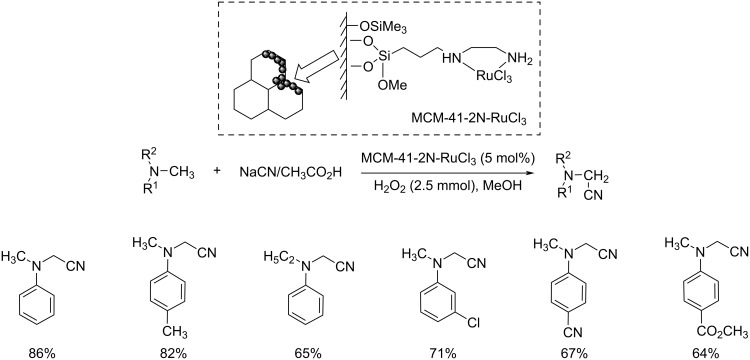
Cyanation of tertiary amines using immobilized MCM-41-2N-RuCl_3_ as the catalyst.

The first report on an MCM-41-immobilized *N*-alkylethylenediamine Ru(III) complex (MCM-41-2N-RuCl_3_) catalyzed oxidative cyanation of tertiary amines was achieved by Cai et al. [31]. The optimized conditions were MCM-41-2N-RuCl_3_ (5 mol %), NaCN (1.2 mmol), AcOH (6.0 mmol), H_2_O_2_ (2.5 mmol) in methanol at 60 °C under Ar atmosphere for 4 h (Scheme 6). The reaction proceeded smoothly for *N*-aryl-substituted cyclic tertiary amines including *N*-arylpiperidines and *N*-aryltetrahydroisoquinolines. The high reusability of the catalyst at least seven times further enhanced the importance of this strategy in the field of organic synthesis.

#### 1.2 Cyanation of amines using homogeneous catalysts

**Scheme 7:**
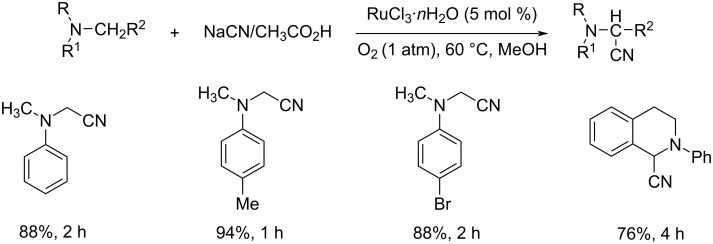
Cyanation of tertiary amines using RuCl_3_·*n*H_2_O as the catalyst and molecular oxygen as oxidant.

An interesting ruthenium-catalyzed oxidative cyanation of tertiary amines using molecular oxygen was reported by Murahashi and co-workers [32]. This RuCl_3_·*n*H_2_O-catalyzed protocol used NaCN in acetic acid as the cyano source, methanol as the solvent under molecular oxygen at 60 °C for 1–2 h (Scheme 7). The extensive substrate scope studies under optimized conditions disclosed that differently substituted *N,N*-dimethylanilines gave the desired products in excellent yields. The reaction was also found suitable for *N*-phenyltetrahydroisoquinoline and delivered the expected product in good yields.

**Scheme 8:**
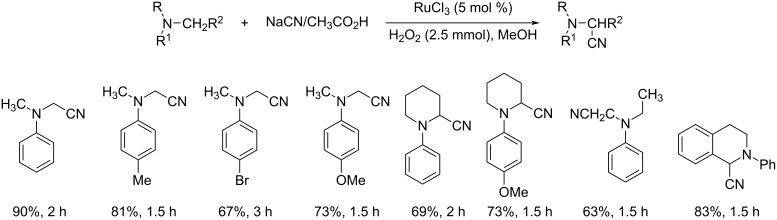
RuCl_3_-catalyzed cyanation of tertiary amines using NaCN/HCN and H_2_O_2_ as oxidant.

Later, the same group reported a novel synthetic pathway for the oxidative cyanation of tertiary amines using sodium cyanide [33]. The optimized conditions for this reaction included the use of 0.05 mmol of RuCl_3_ as the catalyst, 1.2 mmol of NaCN in acetic acid as the cyanide source and 2.5 mmol of H_2_O_2_ as the oxidant in methanol (Scheme 8). Both, substituted *N,N*-dimethylanilines with electron-donating and electron-withdrawing groups were well tolerated in this reaction. The *N*-aryl-substituted cyclic amines such as *N*-arylpiperidine, *N*-aryltetrahydroisoquinoline and *N*-arylpyrrolidine derivatives also reacted well to furnish the desired products in good yields.

**Scheme 9:**
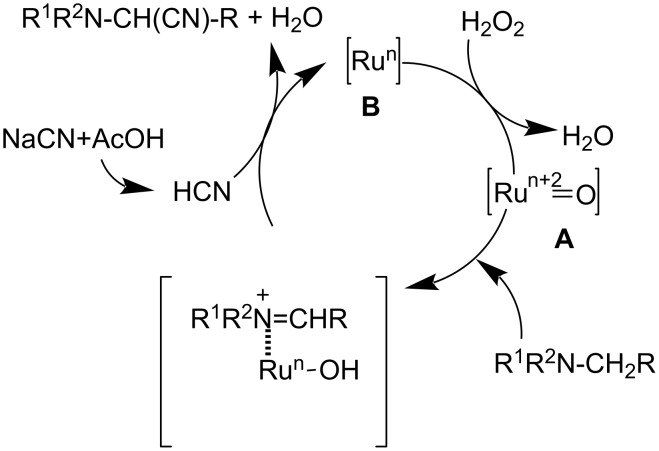
Proposed mechanism for the ruthenium-catalyzed oxidative cyanation using H_2_O_2_.

**Scheme 10:**
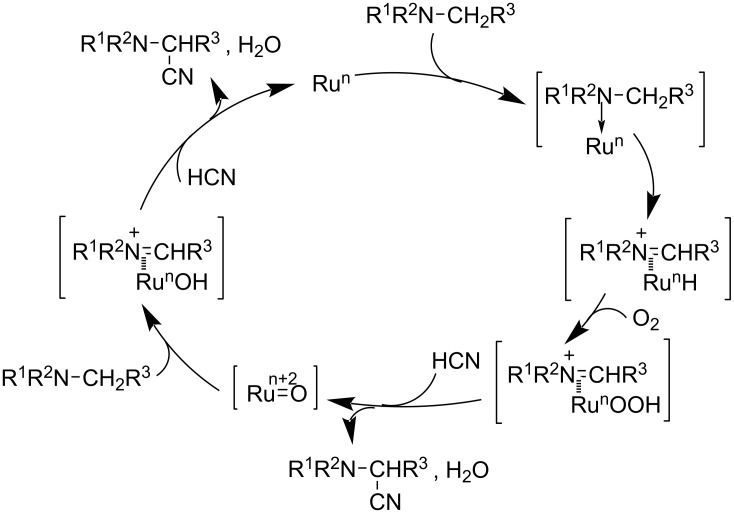
Proposed mechanism for the ruthenium-catalyzed aerobic oxidative cyanation.

In 2008, they described the scope and mechanism of the oxidative cyanation of tertiary amines using H_2_O_2_ and O_2_ [34]. They pointed out the fact that the hydrogen peroxide system was found to be more efficient in catalyzing the cyanation reaction of cyclic amines than the aerobic oxidation system. The catalytic cycle for the hydrogen peroxide system involves the formation of the oxoruthenium species (**A**) and the low-valent ruthenium species (**B**), whereas the aerobic oxidation system includes C–H activation and a subsequent reaction with molecular oxygen (Scheme 9 and Scheme 10). Thus, the authors came to the conclusion that the higher yields of cyanation product obtained in the case of the hydrogen peroxide system was due to the highly reactive oxoruthenium species and low-valence ruthenium species.

**Scheme 11:**
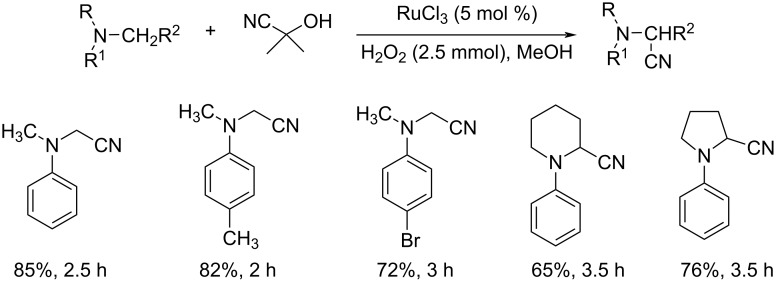
RuCl_3_-catalyzed oxidative cyanation of tertiary amines using acetone cyanohydrin as the cyanating agent.

Sain et al. disclosed a new methodology towards the oxidative cyanation of tertiary amines using Ru as the catalyst (Scheme 11) [35]. In this reaction, the comparatively safer acetone cyanohydrin was utilized as the cyanating agent. Better yields of products were obtained for both electron-rich and electron-deficient tertiary amines. Cyclic amines such as piperidine, tetrahydroisoquinoline derivatives, and pyrrolidine were also tolerated well in this reaction. The use of the non-toxic and inexpensive acetone cyanohydrin makes this method more advantageous compared to the known methods.

### 2 Cyanation of arenes and heteroarenes

#### 2.1 Cyanation of arenes and heteroarenes using heterogeneous catalysts

**Scheme 12:**
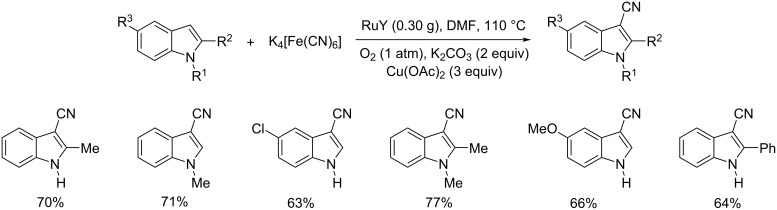
Cyanation of indoles using K_4_[Fe(CN)_6_] as cyano source and Ru(III)-exchanged NaY zeolite (RuY) as catalyst.

In 2012, a novel strategy for the 3-cyanation of indole in the presence of Ru(III)-exchanged NaY zeolite (RuY) was reported [36]. In this reaction K_4_[Fe(CN)_6_] was utilized as the cyano source in DMF at 110 °C. The Cu(OAc)_2_ and O_2_ (1 atm) was found essential for promoting this reaction (Scheme 12). Both electron-rich and electron-deficient indoles afforded the desired products in good yields. The major advantages of this method include high regioselectivity, mild reaction conditions, and reusability of the catalyst.

**Scheme 13:**
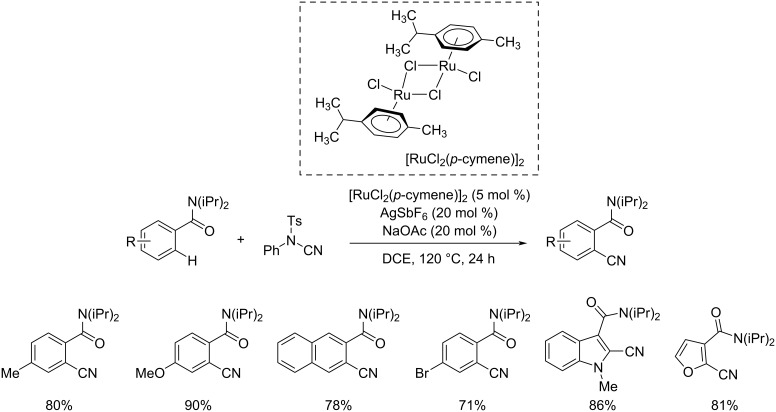
Cyanation of arenes and heteroarenes using a ruthenium(II) catalyst and *N*-cyano-*N*-phenyl-*p*-toluenesulfonamide (NCTS) as cyanating reagent.

**Scheme 14:**
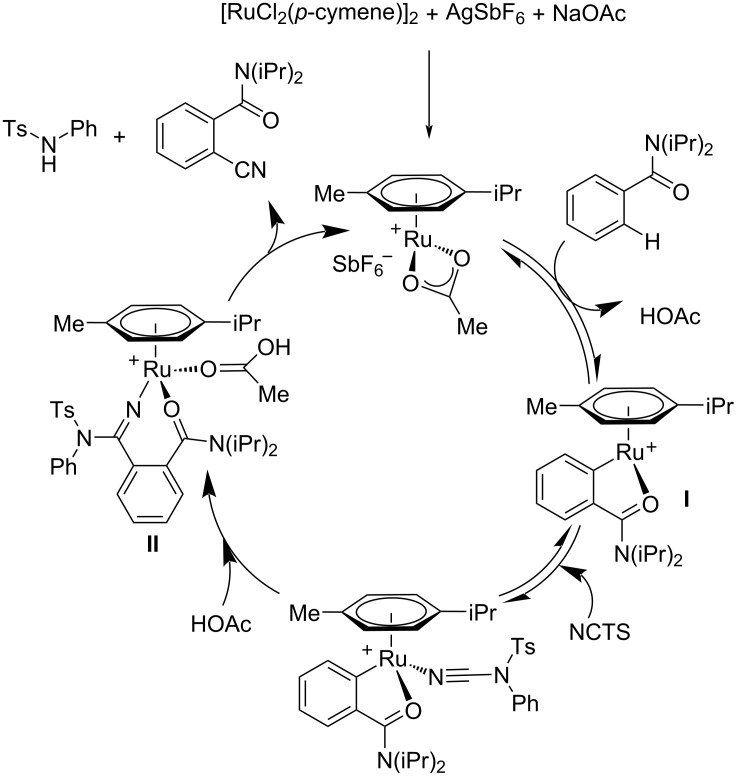
Proposed mechanism for the cyanation of arenes and heteroarenes using ruthenium(II) as catalyst and NCTS as cyanation reagent.

Two years later, in 2014, Ackermann and co-workers disclosed an astonishing protocol towards the cyanation of arenes and heteroarenes using a ruthenium(II) catalyst [37]. This was the first report on a C(sp^2^)–H cyanation reaction using ruthenium as the catalyst. In this reaction the authors utilized the less toxic, environment-friendly, and easily available *N*-cyano-*N*-phenyl-*p*-toluenesulfonamide (NCTS) as the cyanating reagent. The reaction exhibited high chemoselectivity and good functional group tolerance. The optimized conditions for the reaction were [RuCl_2_(*p*-cymene)]_2_ (5 mol %), AgSbF_6_ (20 mol %), NaOAc (20 mol %) in DCE at 120 °C for 24 h (Scheme 13). Differently substituted aromatic amides with a range of functional groups such as fluoro, chloro, bromo, ester etc. were tolerated well in this method. The authors also performed the cyanation of heteroarenes at C-2 and C-3 positions and obtained excellent results. Various heteroarenes such as thiophenes, benzofurans, furans, and indoles were found suitable substrates and afforded the desired products with high chemo- and site-selectivity. A possible mechanism for the reaction was also described. The first step of the catalytic cycle involves the formation of a cationic complex **I** which after coordination and subsequent NCTS insertion is transformed to intermadiate **II**. β-Elimination finally delivers the required product (Scheme 14).

**Scheme 15:**
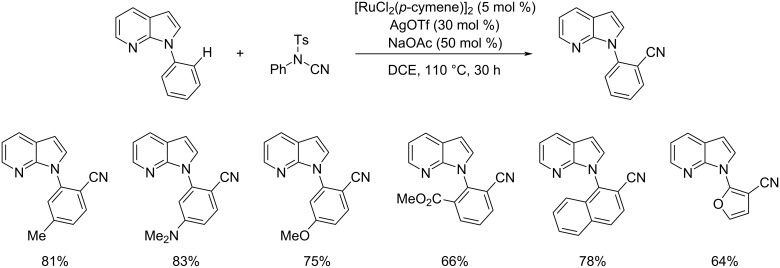
Synthesis of *N*-(2-cyanoaryl)-7-azaindoles.

Later, Deb and co-workers developed a methodology towards the synthesis of *N*-(2-cyanoaryl)-7-azaindoles using [RuCl_2_(*p*-cymene)]_2_/AgOTf/NaOAc as the catalyst system (Scheme 15) [38]. They carried out extensive substrate scope studies and it was pointed out that *N*-aryl-7-azaindoles substituted with electron-donating groups gave better yields of products. Heteroarenes also performed well in this reaction with high reactivity. The authors also investigated the cyanation of differently substituted 7-azaindoles and achieved excellent results. They also succeeded in applying this strategy to *N*-aryl-α-carbolines to synthesize the respective cyanated *N*-aryl-α-carboline products.

### 3 Photocatalyzed cyanation reactions

**Figure 1:**
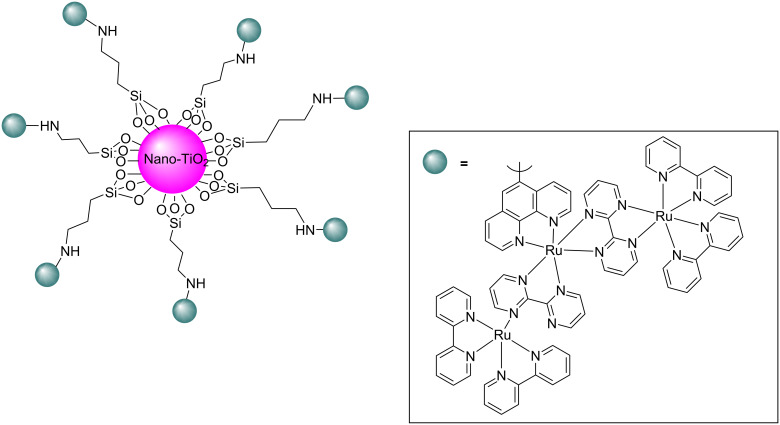
Structure of the TiO_2_-immobilized ruthenium polyazine complex.

Nowadays photocatalysis has emerged as an efficient tool for the synthesis of organic compounds. Jain et al. put forward an oxidative cyanation of tertiary amines using an immobilized heterogeneous ruthenium catalyst (Figure 1) [39]. The optimized conditions comprised sodium cyanide (NaCN) (1.2 mmol) in acetic acid as cyanide source, molecular oxygen as the oxidant, and TiO_2_-immobilized ruthenium(II) polyazine complex as the heterogeneous photoredox catalyst in methanol at room temperature (Table 1). The substrate scope studies revealed a better reactivity of aromatic tertiary amines substituted with electron-donating groups compared to the electron-withdrawing ones. The *N*-aryl cyclic amines including *N*-phenylpiperidine, *N*-phenyltetrahydroisoquinoline, and *N*-phenylpyrrolidine smoothly reacted to deliver the required α-aminonitriles in good yields. Under the optimized conditions, aliphatic tributylamine failed to achieve the desired product (Table 1, entry 5). However, tertiary amines with a benzyl group reacted very slowly and afforded the expected product in moderate yield (Table 1, entry 6). The reason for the incompatibility of tributylamine towards this method has yet to be explored.

**Table 1:** Cyanation of tertiary amines using heterogeneous photoredox catalyst.

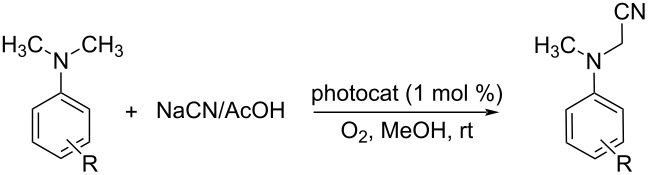				

entry	reactand	product	time (h)	yield (%)

1	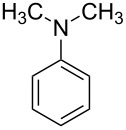	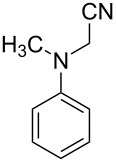	4	96
2	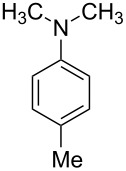	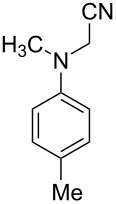	4	91
3	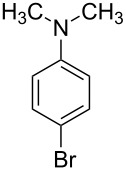	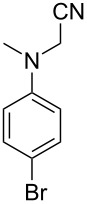	4	85
4	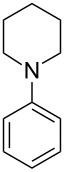	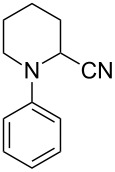	4.5	88
5	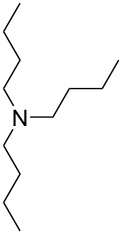	–	48	–
6	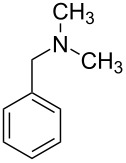	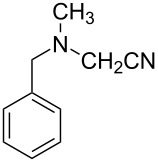	30	48

**Scheme 16:**
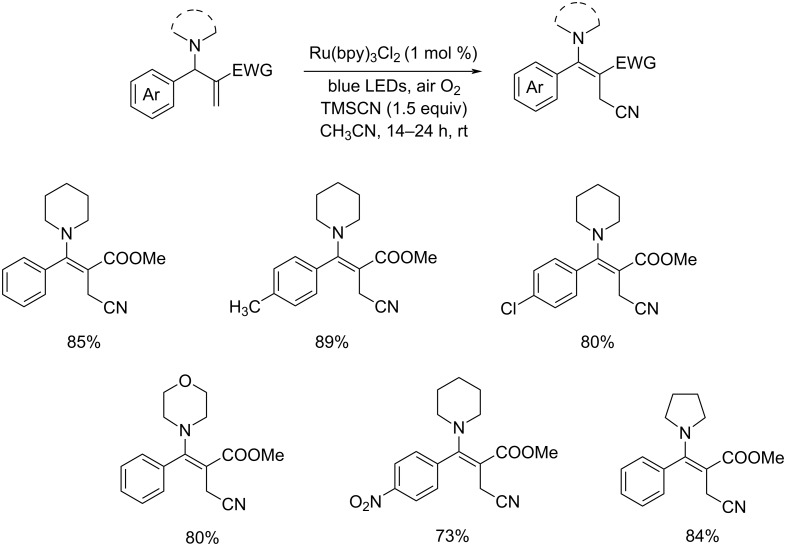
Visible-light-induced oxidative cyanation of aza-Baylis–Hillman adducts.

A synthesis of allylic cyanides via visible-light-mediated oxidative cyanation of aza-Baylis–Hillman adducts was developed by Yadav and co-workers [40]. The cyanating agent employed was TMSCN in acetonitrile (Scheme 16). The aza-BH adducts with electron-donating groups gave higher yields in comparison with electron-deficient ones. Mild reaction conditions, readily available reagents, lower catalyst loading, and the use of cost effective atmospheric oxygen and visible light are the most attractive characteristics of this reaction.

**Scheme 17:**
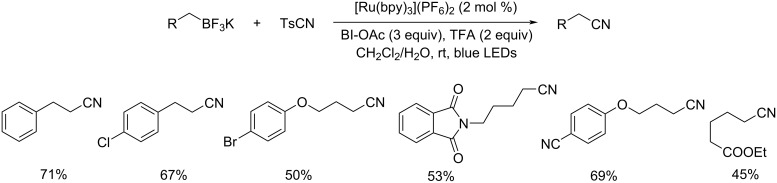
Synthesis of 1° alkyl nitriles using [Ru(bpy)_3_](PF_6_)_2_ as the photocatalyst.

**Scheme 18:**
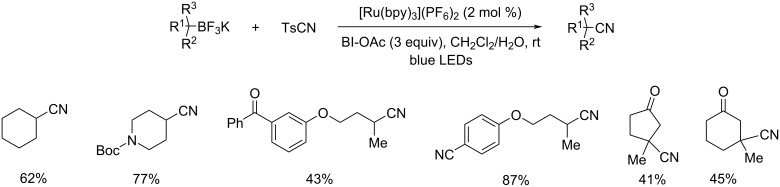
Synthesis of 2° and 3° alkyl nitriles using [Ru(bpy)_3_](PF_6_)_2_ as the photocatalyst.

Xu et al. in 2016 discussed the deboronative cyanation of alkyltrifluoroborates using [Ru(bpy)_3_](PF_6_)_2_ as the photoredox catalyst [41]. This method provides an efficient pathway to 1°, 2°, and 3° alkyl nitriles using *p*‐toluenesulfonyl cyanide (TsCN) in CH_2_Cl_2_/H_2_O under visible-light irradiation (Scheme 17). 1‐Acetoxy‐1,2‐benziodoxol‐3‐(1*H*)‐one (BI‐OAc) was chosen as the oxidant and TFA as the additive in this method. A lower reactivity was observed for 2° and 3° alkyltrifluoroborates under the optimized conditions. The authors were able to improve the product yield by increasing the amount of TsCN and avoiding the use of additive (Scheme 18). A wide variety of functional groups such as esters, cyano, amides, ethers, ketones, alkynes, and halides were compatible with this strategy.

**Scheme 19:**
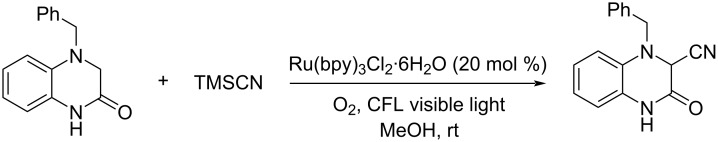
Photoredox cross coupling reaction.

A photoredox-catalyzed oxidative coupling of 4-alkyl-3,4-dihydroquinoxalin-2(1*H*)-ones with nucleophiles was reported by Hong and co-workers [42]. The reaction was performed using 20 mol % of Ru(bpy)_3_Cl_2_**^.^**6H_2_O in methanol under CFL light irradiation at room temperature (Scheme 19). They were able to synthesize the cyano derivative by utilizing TMSCN as the cyanating medium, and obtained the desired product in 74% yield.

**Scheme 20:**
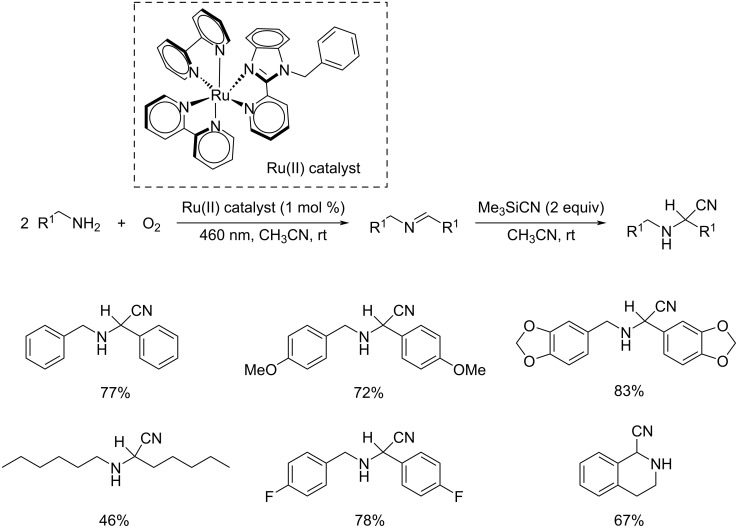
Synthesis of α-amino nitriles from amines via a one-pot strategy.

**Scheme 21:**
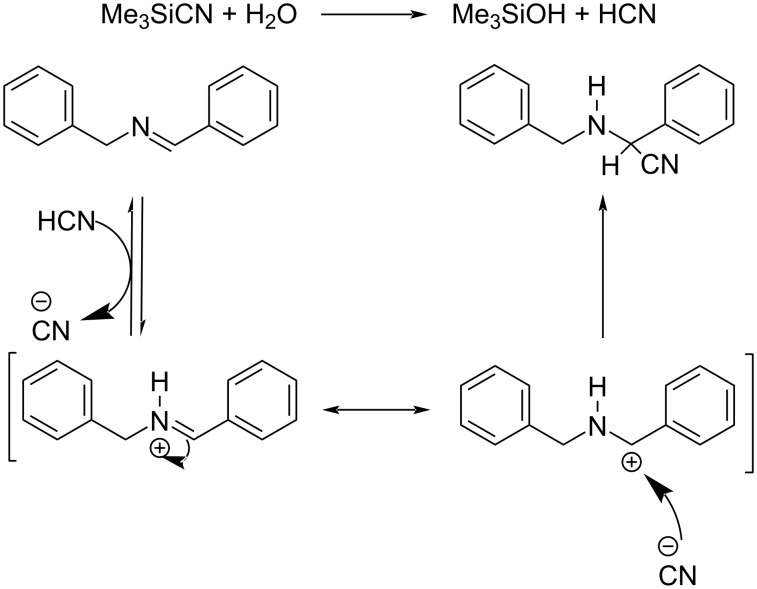
Proposed mechanistic pathway for the cyanation of the aldimine intermediate.

Espino and co-workers developed and characterized a variety of dicationic Ru(II) polypyridyl complexes with 2-(pyridyl)benzimidazole or its N-alkylated derivatives as the ancillary ligands (N^N) [43]. The prepared Ru(II) derivatives were found efficient in the synthesis of α-amino nitriles from amines via a one-pot strategy. This synthetic pathway comprises two consecutive reactions including photooxidation of the amine and the cyanation of resultant aldimine intermediate to afford the α-amino nitriles (Scheme 20). This reaction worked well under eco-friendly conditions with low catalyst loading, and utilizing O_2_ as green oxidant to give excellent yields of the products. They also disclosed the efficiency of these Ru(II) complexes in the photooxidation of primary and secondary amines. Moreover, 1-hexylamine worked well in this reaction affording the desired product in moderate to good yields. The efficient transformation of 1-hexylamine proved that this reaction was even applicable to amines with non-activated α-H atoms. The proposed mechanism for the cyanation step is depicted in Scheme 21.

Chemical synthesis usually needs labor-intensive and tiresome procedures. One of the approaches to overcome these challenges is to perform the reactions in flow [44]. The major advantages of this approach include predictable reaction scale-up, high reproducibility and yields, lower catalyst loading, high product purity, and excellent selectivity.

**Scheme 22:**
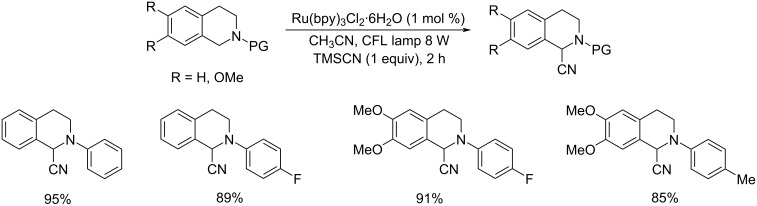
Strecker-type functionalization of *N*-aryl-substituted tetrahydroisoquinolines under flow conditions.

A visible-light-promoted Strecker-type functionalization of *N*-aryl-substituted tetrahydroisoquinolines under flow conditions was reported (Scheme 22) [45]. The cyanating agent utilized was trimethylsilyl cyanide (TMSCN) in acetonitrile solvent under CFL light irradiation.

### 4 Miscellaneous

**Scheme 23:**
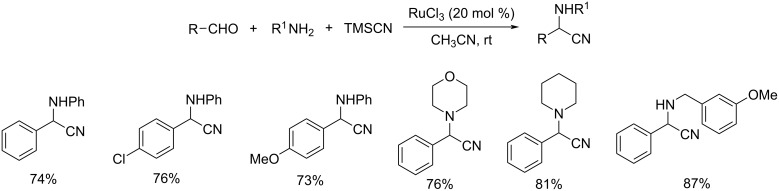
One-pot synthesis of α-aminonitriles using RuCl_3_ as catalyst.

An efficient methodology for the synthesis of α-aminonitriles via the one-pot coupling of aldehydes, amines and trimethylsilyl cyanide was reported [46]. This reaction was catalyzed by RuCl_3_ and used acetonitrile as solvent (Scheme 23). Both aromatic and aliphatic aldehydes performed well and gave the desired products in good yields. At the same time ketones were found less active in this strategy. The operational simplicity and high yield of products are the prominent characteristics of this reaction.

**Scheme 24:**
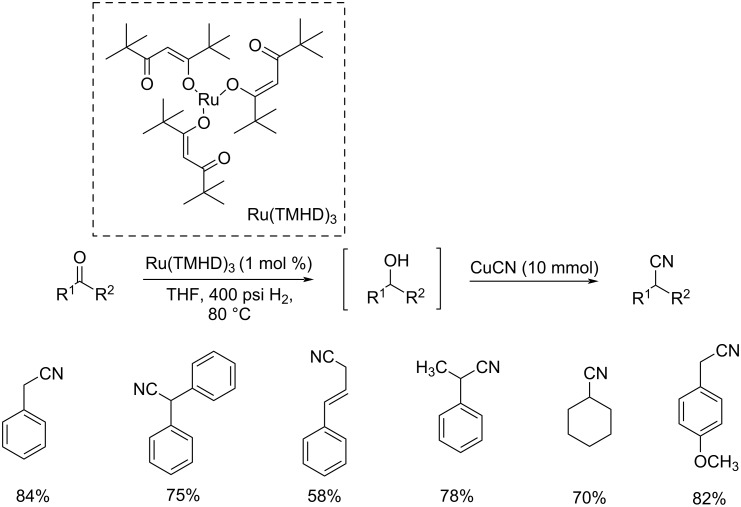
Synthesis of alkyl nitriles using (Ru(TMHD)_3_) as the catalyst.

In 2008, Bhanage et al. developed a novel methodology for the synthesis of alkyl iodides/nitriles using ruthenium tris(2,2,6,6-tetramethyl-3,5-heptanedionate) (Ru(TMHD)_3_) as the catalyst (Scheme 24) [47]. This catalyst was found highly efficient in the hydrogenation, iodination, and cyanation reaction of carbonyl compounds under environmentally benign conditions. This method provides an efficient synthetic route towards alkyl iodides and nitriles in one pot. Carbonyl compounds such as cinnamaldehyde, acetophenone, and cyclohexanone etc. were well tolerated in this reaction and afforded the corresponding nitriles in moderate to good yields.

**Scheme 25:**
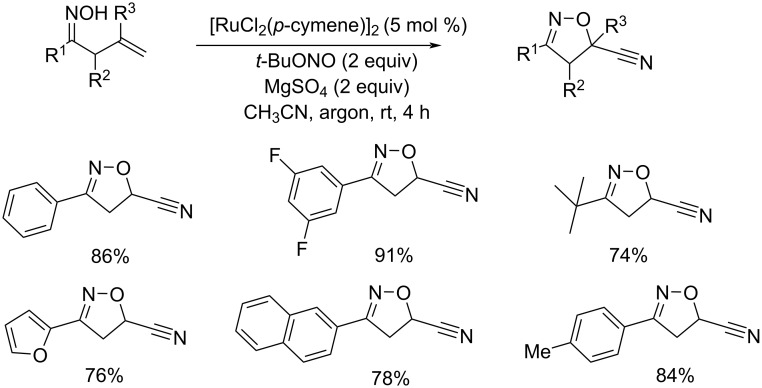
Synthesis of cyanated isoxazolines from alkenyl oximes catalyzed by [RuCl_2_(*p*-cymene)]_2_ in the presence of *tert*-butyl nitrite.

**Scheme 26:**
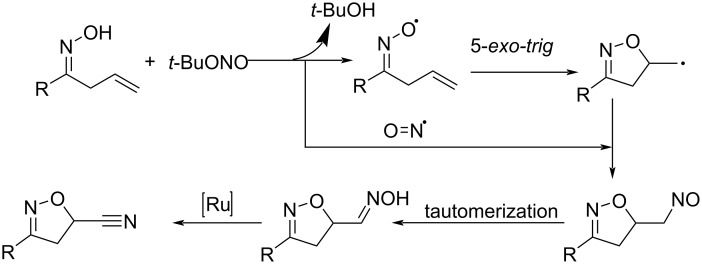
Proposed mechanism for the synthesis of cyanated isoxazolines from alkenyl oximes.

Zhang and co-workers developed an efficient methodology for the synthesis of cyanated isoxazolines from the corresponding alkenyl oximes under mild reaction conditions (Scheme 25) [48]. The dual role of *tert*-butyl nitrite as oxidant and as a nitrogen source further enhanced the significance of this method. [RuCl_2_(*p*-cymene)]_2_ was identified as the best choice of catalyst. Differently substituted alkenyl oximes with aryl, heteroaryl, and alkyl substituents performed well in this reaction. The major advantage of this method is the formation of C–O and C≡N bonds in a single-step. The proposed mechanism is depicted in Scheme 26.

**Scheme 27:**
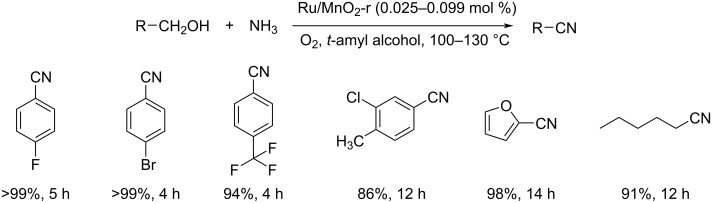
Oxidative cyanation of differently substituted alcohols.

Xiao and co-workers developed an environmentally benign strategy for the oxidative cyanation of differently substituted alcohols using a manganese oxide nanorod-supported ruthenium catalyst (Scheme 27) [49]. They also evaluated the efficiency of other metals such as Au, Pt, Pd, Rh, Ag, and Fe and found that they were less efficient compared to Ru. The benzylic alcohols showed higher reactivity than aliphatic alcohols towards this methodology. Moreover, this protocol worked well for differently substituted heterocyclic alcohols and afforded the products in excellent yields. The authors also conducted various experimental and theoretical studies to analyze the reaction mechanism. The proposed mechanism begins with the oxidative dehydrogenation of the alcohol to afford the aldehyde which undergoes condensation with ammonia to give the corresponding imine. Finally, oxidative dehydrogenation results in the formation of the nitrile.

## Conclusion

This review summarizes the recent progress in ruthenium-catalyzed cyanation reactions. Due to the wide application of nitrile compounds in pharmaceutical and biological fields, cyanation reactions have achieved significant progress in recent times.

Transition-metal-catalyzed cyanation reactions have emerged as an alternative approach to conventional cyanation strategies. Nowadays ruthenium has gained much acceptance owing to its wide range of oxidation states and ability to form a large number of complexes. From our discussion it is evident that the most commonly used cyanating reagents include highly toxic compounds such as K_4_[Fe(CN)_6_], NaCN, CuCN, TMSCN etc. However, some of the reported works involve the use of safer and greener cyanation sources like NCTS, acetone cyanohydrin, ethyl cyanoformate etc.

Our studies disclosed that many of the reports were mainly focusing on the oxidative cyanation of tertiary amines. Extensive research was carried out in the cyanation of tertiary amines using different oxidants such as O_2_, H_2_O_2_, and TBHP. Ruthenium was also found to be effective in the cyanation of arenes and heteroarenes with high functional group tolerance. Some of the suggested methods utilized photoredox catalysts and thus offer new avenues towards greener and economical nitrile synthesis.

In spite of these advancements, the use of toxic cyanating agents like NaCN in almost all the methods remains as a limitation in this area. The use of non-toxic and environment friendly cyanating agents such as NCTS, acetone cyanohydrin, ethyl cyanoformate etc needs more attention in future. Scientists can also focus more on cyanation reactions using photoredox catalyst.

The larger availability of reactive complexes of ruthenium and the high reusability and easy separation procedures of heterogeneous catalysts have proven the fact that this metal serve as highly effective catalyst for a wide range of organic transformations. It will surely motivate the scientific community to develop more environmentally benign cyanation reactions using ruthenium in future.
